# Comparative efficacy and acceptability of sleep interventions for children and adolescents with autism spectrum disorders: a protocol for a systematic review and network meta-analysis

**DOI:** 10.1186/s13643-026-03143-8

**Published:** 2026-02-27

**Authors:** Masatsugu Sakata, Edoardo G. Ostinelli, Ryuichiro Yamamoto, Hitomi Oi, Shino Kikuchi, Rie Toyomoto, Shun Nakajima, Kei Ohashi, Akane Nogimura, Rie Yamada, Laurie McLay, Toshi A. Furukawa, Yukiyo Nagai, Atsurou Yamada

**Affiliations:** 1 Department of Neurodevelopmental Medicine, Nagoya City University Graduate School of Medicine, Nagoya, Japan; 2 Department of Health Promotion and Behavioral Sciences, Kyoto University Graduate School of Medicine/School of Publich Health, Kyoto, Japan; 3 International Institute for Integrative Sleep Medicine (WPI-IIIS), Tsukuba Institute for Advanced Research (TIAR), University of Tsukuba, Tsukuba, Japan; 4 Department of Psychiatry, University of Oxford, Oxford, UK; 5 Oxford Precision Psychiatry Lab, National Institute for Health Research Oxford Health Biomedical Research Centre, University of Oxford, Oxford, UK; 6 Oxford Health NHS Foundation Trust, Oxford, UK; 7 College of Sociology, Department of Psychology and Humanities, Edogawa University, Nagareyama, Chiba Japan; 8 Department of Psychological Sciences, University of Human Environments, Matsuyama, Japan; 9 Core Laboratory, Center for Psycho-Oncology and Palliative Care, Nagoya City University Graduate School of Medical Sciences, Nagoya, Japan; 10 Department of Health Policy, National Center for Child Health and Development, Tokyo, Japan; 11 Faculty of Health, Child Well-Being Research Institute, University of Canterbury, Christchurch, New Zealand; 12 Kyoto University Office of Institutional Advancement and Communications, Kyoto, Japan

**Keywords:** Developmental disorders, Sleep disturbances, Melatonin, Orexin, Behavior therapy, Chronotherapy, Light therapy, Matless technology, Weighted blanket

## Abstract

**Background:**

Children and adolescents with autism spectrum disorder (ASD) frequently experience sleep problems. Although various pharmacological, behavioral, and physical interventions have demonstrated efficacy in improving sleep among children with ASD, the relative effectiveness of these interventions remains unclear.

**Methods:**

We will conduct a systematic literature search to identify randomized controlled trials that evaluate the efficacy of pharmacological (e.g., melatonin), psychological (e.g., cognitive behavioral therapy), and physical (e.g., bright light therapy) interventions for sleep problems in children with ASD. We will search PubMed, PsycINFO, Cochrane CENTRAL, major trial registries, and regulatory agency websites. We will assess the Cochrane Risk of Bias 2.0 (RoB 2.0) tool for primary outcome and the Risk Of Bias due to Missing Evidence in Network meta-analysis (ROB-MEN) tool for the bias due to missing network evidence. A network meta-analysis (NMA) will be performed to compare the included interventions. The primary outcome will be sleep onset latency, while secondary outcomes will include other sleep variables, all-cause dropouts, and sleep disturbances assessed using standardized measures. We will assess confidence in NMA(CINeMA).

**Discussion:**

Our NMA aims to provide evidence-based insights into the effectiveness of sleep interventions for clinicians, children with ASD, and their caregivers. This information will help guide treatment decisions and improve the quality of life for children with ASD and their families.

**Systematic review registration:**

PROSPERO CRD42024592795.

**Supplementary Information:**

The online version contains supplementary material available at 10.1186/s13643-026-03143-8.

## Background

Sleep disturbances represent one of the most commonly reported problems among children and adolescents (henceforth, children) with autism spectrum disorder (ASD), affecting between 37 to 93% of children [1]. Children and adolescents with ASD tend to experience higher rates of sleep problems such as bedtime resistance, prolonged sleep onset latency, short sleep duration, sleep-related anxiety, and daytime sleepiness compared to their non-autistic peers [2]. These sleep problems often persist into adulthood [3]. In addition, impaired sleep quality is associated with the severity of social dysfunction, sensory sensitivity, and internalizing and externalizing behavior problems in children with ASD [4]{Du, 2023, Neurometabolite levels in the brains of patients with autism spectrum disorders: A meta-analysis of proton magnetic resonance spectroscopy studies (N = 1501)}.

Recent systematic reviews demonstrate the efficacy of melatonin [5] and behavioral interventions such as stimulus control, extinction, or reinforcement for sleep problems among children with ASD [6]. Emerging evidence also demonstrates the effectiveness of cognitive behavioral therapy (CBT) for these children [7]. Improvement in sleep following intervention has been shown to result in secondary improvement in stereotypic behavior, internalizing and externalizing behavior problems, and quality of life [8], underscoring the importance of effective sleep intervention.

The American Academy of Neurology (AAN) developed a comprehensive practice guideline based on its systematic review, recommending the combination of melatonin and cognitive behavioral therapy (CBT) or melatonin alone as interventions for bedtime resistance, sleep onset latency, sleep continuity, and total sleep time (Williams Buckley et al., 2020). However, the comparative efficacy and acceptability of each intervention is unclear. The recommendations of the AAN were based on a series of pairwise meta-analyses of each intervention compared with control condition and, therefore, cannot inform the relative effects of each intervention approach. A recent network meta-analysis focusing on the general pediatric patient population showed that light therapy is effective for sleep onset latency (SOL), an evidence-based psychological intervention (EBPI) that includes CBT and parental intervention for wake-after-sleep onset (WASO), and EBPI plus light therapy for total sleep time (TST) compared to control condition [9]. Still, there are several challenges in applying these results to children with ASD. First, the comparative effectiveness of different sleep interventions for the ASD population remains unclear. Second, psychological and behavioral interventions have distinct therapeutic mechanisms and may have varying effects depending on the child's characteristics. As such, they should be separate into one category to inform clinical decision-making pragmatically. It is also inappropriate to classify the various control conditions used in these studies into one node because different control conditions, e.g. placebo, treatment as usual or waiting list, lead to different efficacy estimates [10]. Third, since 2020, there has been an exponential increase in clinical trials of sleep interventions for people with ASD, warranting analyses and syntheses that reflect recent data in the 2020 s [11, 12].

To address these issues, we hereby plan a network meta-analysis (NMA) of sleep intervention for children and adolescents with ASD.

### Aim

We will investigate the comparative efficacy and acceptability of pharmacological, psychological, behavioral, and physical interventions for disrupted sleep in children and adolescents with autism spectrum disorder (ASD).

## Methods

The protocol follows the Preferred Reporting Items for Systematic Reviews and Meta-Analyses Protocols (PRISMA-P) [13] and extension statement for network meta-analysis [14] where relevant for protocols. We prospectively registered this protocol to PROSPERO (CRD42024592795).

## Data sources

### Criteria for considering studies for this review

#### Study design

We will incluonde all randomized ctrolled trials of sleep-focused interventions compared with other types of intervention or control conditions for children and adolescents with ASD.

#### Participants

Participants will be children and adolescents under 24 years of age, of any gender, who meet diagnostic criteria for ASD or meet the thresholds of clinically relevant ASD traits using a standardized screening scale. Studies allowing participants with comorbid conditions (e.g., epilepsy, intellectual disabilities, attention deficit hyperactivity disorders, major depressive disorders, anxiety disorders, and obsessive–compulsive disorders) will be included. Still, we will test the influence of their inclusion in sensitivity analyses.

#### Interventions and controls

We will select interventions from the practice guidelines of the American Academy of Neurology [15] and a recent network meta-analysis on sleep intervention for general pediatric populations [9]. All pharmacological, psychological, behavioral, and physical interventions will be considered eligible. Pharmacological interventions (i.e., medications) will include oral melatonin, orexin receptor antagonists, antipsychotics, and other hypnotic medications used to treat sleep disturbances. Psychological and behavioral interventions refer to those that are underpinned by psychological theories and/or principles of applied behavior analysis and will include cognitive behavioral therapy for sleep problems, parent education, parental training, and chronotherapy. Physical interventions involve modification to the physical environment to primarily result in physiological (as opposed to behavioral) changes, including bright light therapy, Sound-to-Sleep mattress technology, and weighted blankets. We will treat any combined intervention (e.g., CBT + melatonin) as a distinct node in the network meta-analysis if it is evaluated as a separate arm in the original study. This applies to both within-category (e.g., CBT + parent education) and cross-category (e.g., behavioral + pharmacological) combinations.

The control conditions will include a pill placebo, attention/psychological placebo, no treatment, treatment as usual, and waiting list. When the placebo, no treatment or treatment as usual are used while waiting to receive intervention, such arms will be classified as the waiting list. We will include any psychological and behavioral intervention format, including self-help learning with or without guidance, internet or human-administered programs, face-to-face, hybrid or remote, and group or individual. Table 1 shows a list of included interventions and control conditions.

**Table 1:** Definition of interventions and control conditions

Names	Description
**Pharmacological intervention**	
Oral melatonin	A hormone naturally produced by the pineal gland that is often used as a supplement to regulate circadian rhythms and manage sleep disorders. We will include melatonin receptor agonists such as ramelteon
Orexin receptor antagonist	A class of drugs that function by inhibiting the activity of orexins, neurotransmitters that play a key role in maintaining wakefulness
Other hypnotic medication	Other medications that purpose to improve sleep such as benzodiazepines and trazodone
**Psychological and behavioral intervention**	
Cognitive behavioral therapy	Structured programs help individuals identify and replace thoughts and behaviors that maintain sleep problems with habits that promote sound sleep, including sleep restriction, stimulus control, cognitive restructuring, sleep/wake rescheduling, and mindfulness. These programs may include written, digital, or in-person information delivered individually or in groups
Parent education and training	Provide parent psychoeducation about child sleep, including sleep hygiene, sleep scheduling, and contingency management. This may include written, digital, or in-person information delivered in individual or group format
Chronotherapy	Chronobiological intervention gradually shifts sleep and wake times to adjust the sleep schedule to align with desired or socially acceptable times
**Physical or environmental interventions**	
Bright light therapy	Chronobiological intervention that adjusts sleep time by stimulating cells in the retina, which connects to the hypothalamus, the brain region controlling human circadian rhythms
Adapted mattress technology	Mattress technology uses embedded acoustic resonators to transmit gentle sound waves, aiming to improve sleep quality
Weighted blanket	Blankets, often filled with materials like plastic pellets or glass beads, to apply gentle, deep-pressure stimulation across the body to promote relaxation and sleep onset
**Control conditions**	
Pill placebo	Often, sugar pills or treatments without active ingredients can elicit therapeutic effects through the power of belief and expectation and are used in clinical trials
Psychological/attention placebo	Control conditions that could generate expectation intervention effects for trial participants, such as health education, sleep hygiene, or relaxation training
Usual care	Typical continuous care or treatment in the context of each healthcare system
Waiting list	Conditions that provide delayed intervention after outcome collection

### Search methods for identification of studies

We will conduct a comprehensive literature search in PubMed, PsycINFO, Cochrane CENTRAL, major trial registries, and regulatory agency websites with the American Academy of Neurology's revised search strings (Table 2) until the most current date. A medical librarian from Nagoya City University Kawasumi Library, with experience in supporting systematic reviews, peer-reviewed our search strategy in accordance with the PRESS (Peer Review of Electronic Search Strategies) guideline [16] (see Supplementary information).

**Table 2:** Search strings for PubMed, Cochrane CENTRAL, and PsycINFO

PubMed	(((autis*[tiab] OR asperger*[tiab] OR "pervasive development* disorder*"[tiab] OR "Child Development Disorders, Pervasive"[Mesh]) AND ("Sleep Wake Disorders"[Mesh] OR sleep*[tiab] OR circadian*[tiab])) AND (randomized controlled trial[pt] OR controlled clinical trial[pt] OR randomized[tiab] OR placebo[tiab] OR "drug therapy"[sh] OR randomly[tiab] OR trial[tiab] OR groups[tiab])) AND (child*[tiab] OR adolescent*[tiab] OR youth*[tiab] OR "young adult*"[tiab] OR "Child"[Mesh] OR "Adolescent"[Mesh] OR "Young Adult"[Mesh])
Cochrane CENTRAL	((autis* OR asperger* OR ("pervasive developmental disorders" OR "pervasive developmental disorder" OR "pervasive development disorders" OR "pervasive development disorder")):ti,ab,kw OR [mh "Child Development Disorders, Pervasive"]) AND ([mh "Sleep Wake Disorders"] OR (sleep* OR circadian*):ti,ab,kw) AND ([mh child] OR [mh Adolescent] OR [mh "Young Adult"] OR (child* OR adolescent* OR youth* OR (young NEXT adult*)):ti,ab,kw))
PsycINFO	((autis* OR asperger* OR "Pervasive Developmental Disorder" OR "Autism Spectrum Disorder") AND (sleep* OR circadian* OR insomnia) AND (randomized controlled trial OR controlled clinical trial OR randomized OR placebo OR "drug therapy" OR trial OR groups) AND (child* OR adolescent* OR youth* OR "young adult*"))

We will use a combination of index and free terms relating to sleep interventions and autism spectrum disorder with filters for randomized clinical trials. We will impose no date, language, or publication status restriction and include dissertations, conference abstracts, and trial registries. We will check the reference lists of identified studies and review articles for additional potentially eligible records.

### Data collection and analysis

#### Selection of studies

Two reviewers will independently screen titles and abstracts from the potential studies, classify them as “retrieve” vs. “not retrieve,” and retrieve the full-text publications for the studies identified as potentially relevant. Two reviewers will screen the full text independently and select eligible studies that meet the inclusion criteria. We will resolve disagreements through discussion between pairs or consult with a third reviewer if necessary. We will calculate the kappa for inter-rater reliability of full-text screening. We will identify duplicate publications. We will record details in a PRISMA flow diagram. We will utilize Covidence to facilitate both the title and abstract screening as well as the full-text screening processes.

#### Data items

Two reviewers will independently extract the data from each study's eligible arms in the Covidence. Any disagreement will be resolved through discussion or with a third person if necessary. We will abstract the following information.

1. Characteristics of the studies
  We will extract trial registration ID, number of arms, treatment or control arm, number of each arm, year of publication, country, study site (single or multi-center), study design (individual or cluster), population characteristics (mean age, number of female participants), the definition of ASD, intervention type(pharmacological, psychological and behavioral, or physical), the definition of inclusion, intervention (content, delivery format, duration), duration of follow-up.
2. Risk of bias
  Two reviewers will assess the risk of bias for the primary outcome in each domain of the Cochrane Risk of Bias 2.0 tool (RoB2) [17]. The RoB2 tool is specifically designed for randomized controlled trials and evaluates five key domains of potential bias: (1) bias arising from the randomization process, (2) bias due to deviations from intended interventions, (3) bias due to missing outcome data, (4) bias in the measurement of the outcome, and (5) bias in the selection of the reported result. Each domain will be assessed using signaling questions that guide the reviewers in categorizing the risk of bias as “low risk,” “some concerns,” or “high risk.” These domain-specific judgments will then be used to determine the overall risk of bias for each study.
  If disagreements arise between the two reviewers during the assessment process, a structured approach will be taken to resolve them. The reviewers will discuss the points of contention, referencing the RoB2 guidance document and study-specific information to reach a consensus. In cases where consensus cannot be achieved, a third reviewer with expertise in systematic reviews and bias assessment will be consulted to make a final determination.
  To ensure the reliability of the assessments, inter-rater agreement will be calculated using Cohen’s kappa statistic. This statistical measure will provide an objective evaluation of the consistency between the reviewers’ judgments and highlight any potential areas where further refinement of the assessment process might be required.
  In addition to assessing the risk of bias at the study level, we will evaluate bias at the network level using the Risk of Bias due to Missing Evidence in Network Meta-Analysis (ROB-MEN) tool [18]. The ROB-MEN tool addresses the risk of bias introduced by missing studies or network connections in the meta-analysis. It evaluates whether the evidence available in the network is sufficiently robust and representative to support reliable conclusions. By combining RoB2 assessments with ROB-MEN evaluations, we aim to ensure a comprehensive and transparent appraisal of bias that enhances the credibility and robustness of our findings.
3. Data to calculate effect sizes
  We will calculate the between-group standardized mean difference (SMD) by dividing the differences in the change score from baseline to post-intervention of the continuous outcomes by the observed SD of the endpoint scores (preferred) or by dividing the differences in the endpoint scores by the observed SD of the endpoint scores. We will calculate the odds ratio (OR) for all-cause dropouts from the study.

### Primary outcome and secondary outcomes

The primary outcome is sleep onset latency (SOL) measured by sleep diary at short-term (post-intervention) periods. There are various possible indicators for sleep problems in ASD. However, we consider difficulty falling asleep to be an indicator that covers a wide range of problems, including insomnia, sleep resistance, and delayed sleep phase. We prioritize patients' self-report SOL and other reports if patients' self-reports are not available.

Secondary outcomes include acceptability, defined as all-cause dropouts from the study, wake after sleep onset (WASO), total sleep time (TST), waking time (WT), and other sleep disturbances assessed by standardized measures at a short-term period. We define sleep disturbance widely, including insomnia, circadian sleep phase problems, and sleep resistance or phobias. Sleep disturbances should be evaluated using validated scales such as the Sleep Disturbance Scale for Children (SDSC) [19], the Children's Sleep Habits Questionnaire (CSHQ [20]), the Children’s Report of Sleep Pattern-Sleepiness Scale (CRSPSS [21]) and the Insomnia Severity Index (ISI) [22].

We investigate the effects of intervention for all sleep variables(SOL, WASO, TST, and WT) at the long-term periods(up to six-month follow-up). To maintain interpretability and avoid bias due to time-point heterogeneity, short-term and long-term outcomes will be analyzed separately and not pooled in the same analysis.

We will calculate SMD as reported by the original authors, who followed the intention-to-treat principle as closely as possible. We will calculate the OR by assuming all non-reported cases as dropouts.

### Statistical analyses

We will use R (latest version, R Foundation, Vienna, Austria) (R Core Team, 2020) package “NMA” [23], which implements frequentist contrast-based network meta-analysis methods, for primary analyses. The package is publicly available at https://cran.r-project.org/web/packages/NMA/.

We assume a random-effect model considering clinical and methodological heterogeneities in intervention effects among studies. We will create a network diagram for each outcome and examine the network's transitivity, inconsistency, and heterogeneity.

We will examine transitivity in the network by comparing the distribution of important potential effect modifiers across the comparisons, including gender, age, baseline severity of autistic symptoms, and psychiatric and physical comorbidities. We will examine heterogeneities by comparing common τ^2^ of a network meta-analysis against their empirical distributions [24, 25]. In the case of substantive heterogeneity, we will explore their sources through the pre-planned subgroup analyses. We will investigate inconsistency in the network using the local test of inconsistency (the side-splitting test (König et al., 2013)) and the global test of inconsistency (the design-by-treatment inconsistency model [26].

### Certainty of evidence

We will assess the certainty of evidence in network estimates of the primary outcome using Confidence in Network Meta-Analysis(CINeMA [27]). CINeMA evaluates six domains of confidence in network meta-analysis: within-study bias, reporting bias, indirectness, imprecision, and heterogeneity. We will use the developers’ web application to assess CINeMA [28].

### Publication bias

We will assess small study-effect bias by visually inspecting asymmetry in the contour-enhanced funnel plot (all active interventions against the controls, including the treatment as usual and the waiting list) and performing Peters’ test [29].

### Subgroup analyses

We will conduct subgroup analyses and meta-regressions to examine the impact of age on the outcomes, as developmental differences between younger children, adolescents, and young adults with autism spectrum disorder (ASD) may influence treatment response. Participants will be categorized into predefined age groups (e.g., children under 10 years, adolescents aged 10–17 years, and young adults aged 18–24 years), and the results will be compared to assess age-related effect modifications.

To ensure credibility, we will apply the ICEMAN tool to evaluate the plausibility, consistency, independence, and robustness of the observed subgroup effects [30]. We will verify that findings are supported by prior evidence, consistent across studies, free from confounding by other modifiers, and adequately powered for analysis. Results will be presented as pooled effect estimates with confidence intervals, with meta-regressions assessing age as a continuous variable for deeper insights. Findings will be reported in line with PRISMA and ICEMAN guidelines to ensure transparency and reliability, aiding in clinically meaningful conclusions.

### Sensitivity analyses

We will conduct sensitivity analyses by excluding studies with a high risk of bias (RoB2), studies without a clinical diagnosis of ASD, studies with more than 50% of participants having comorbidity, studies not targeted sleep disorders other than insomnia (e.g. circadian rhythm sleep–wake disorders).

We will compare results from the full dataset and the restricted dataset using forest plots to assess the impact of study quality on network estimates.

### Patient and public involvement

There was no patient or public involvement in preparing this study plan.

## Discussion

Our systematic review and NMA will contribute to the existing body of evidence by synthesizing data on sleep interventions' comparative efficacy and acceptability for ASD children. This work addresses critical gaps in understanding which interventions yield the most significant improvements in sleep outcomes, an area of substantial importance given the high prevalence of sleep disturbances in this population and their impact on overall functioning and quality of life.

By leveraging the power of network meta-analysis, we can integrate direct and indirect evidence to provide a comprehensive overview of the available interventions. The CINeMA framework allows us to systematically evaluate the evidence's certainty, considering domains such as within-study bias, reporting bias, and transitivity. This rigorous approach ensures that our findings are methodologically sound, highly transparent, and interpretable for stakeholders.

Our NMA will empower patients, clinicians, caregivers, and policymakers with robust, evidence-based insights to guide intervention choices tailored to the unique needs of individuals with ASD. By identifying interventions with the highest certainty of evidence and most significant clinical relevance, we aim to improve both short- and long-term outcomes, addressing a critical unmet need in the care of this vulnerable population.

Despite the strengths of this protocol, several limitations should be acknowledged. First, clinical and methodological heterogeneity across included studies may affect the consistency and generalizability of network estimates. Second, the risk of selective or incomplete outcome reporting in original trials may introduce bias into our synthesis. Third, this protocol was developed without direct involvement of patients, caregivers, or other stakeholders, which may limit the relevance of certain outcomes or interpretations. We will address these concerns through sensitivity analyses and transparent reporting practices.

## Supplementary Information

Supplementary Material 1: PRISMA-P 2015 Checklist.

Supplementary Material 2: Appendix 1. *PRESS Guideline* — Search Submission& Peer Review Assessment.

## Data Availability

Not applicable.
