# Supplementary material for: Comparative efficacy and acceptability of sleep interventions for children and adolescents with autism spectrum disorders: a protocol for a systematic review and network meta-analysis
Source: Syst Rev. 2026 Feb 27;15:116. doi: 10.1186/s13643-026-03143-8 (PMC13041137; doi:10.1186/s13643-026-03143-8)
Supplement: Supplementary file 2 — Supplementary Material 2: Appendix 1. PRESS Guideline — Search Submission& Peer Review Assessment. [file 13643_2026_3143_MOESM2_ESM.docx]

# Appendix A

***PRESS Guideline* — Search Submission & Peer Review Assessment**

**SEARCH SUBMISSION: THIS SECTION TO BE FILLED IN BY THE SEARCHER**

| Searcher: Masatsugu Sakata | Email: m.sakata@med.nagoya-cu.ac.jp |  |
| --- | --- | --- |
| Date submitted: | Date requested by: | *[Maximum = 5 working days]* |

**Systematic Review Title:**

Comparative efficacy and acceptability of sleep interventions for children and adolescents with autism spectrum disorders: A systematic review and network meta-analysis

This search strategy is …

| x | My PRIMARY (core) database strategy — First time submitting a strategy for search question and database |
| --- | --- |
|  | My PRIMARY (core) strategy — Follow-up review NOT the first time submitting a strategy for search question and database. If this is a response to peer review, itemize the changes made to the review suggestions |
|  | SECONDARY search strategy— First time submitting a strategy for search question and database |
|  | SECONDARY search strategy — NOT the first time submitting a strategy for search question and database. If  this is a response to peer review, itemize the changes made to the review suggestions |

**Database**

(i.e., MEDLINE,CINAHL…):	*[mandatory]*

PubMed, PsycINFO, Cochrane CENTRAL, major trial registries, and regulatory agency websites

**Interface**

(i.e., Ovid, EBSCO…):	*[mandatory]*

Ovid (only PsycINFO)

**Research Question**

(Describe the purpose of the search)	*[mandatory]*

What is the comparative efficacy and acceptability of pharmacological, psychological, behavioral and physical interventions for disrupted sleep in children and adolescents with autism spectrum disorder?

**PICO Format**

(Outline the PICOs for your question — i.e., Patient, Intervention, Comparison, Outcome, and Study Design — as applicable)

| **P** | Children and adolescents with autism spectrum disorder under 24 years |
| --- | --- |
| **I** | Pharmacological, psychological, behavioral, and physical interventions |
| **C** | Pill placebo, attention/psychological placebo, no treatment, treatment as usual, and waiting list. |
| **O** | Sleep onset latency at post intervention |
| **S** | Randomized Controlled Trial |

**Inclusion Criteria**

(List criteria such as age groups, study designs, etc., to be included) *[optional]*

We will include all randomized controlled trials of sleep-focused interventions compared with other types of intervention or control conditions for children and adolescents with ASD.

**Exclusion Criteria**

(List criteria such as study designs, date limits, etc., to be excluded) *[optional]*

**Was a search filter applied?**

Yes	No

**If YES, which one(s) (e.g., Cochrane RCT filter, PubMed Clinical Queries filter)? Provide the source if this is a published filter.** *[mandatory if YES to previous question* — *textbox]*

Cochrane RCT filter

Other notes or comments you feel would be useful for the peer reviewer? ***[optional]***

Please copy and paste your search strategy here, exactly as run, including the number of hits per line. ***[mandatory]***

**(Add more space, as necessary.)**

**PEER REVIEW ASSESSMENT: THIS SECTION TO BE FILLED IN BY THE REVIEWER**

|  | Reviewer: Minako Tahara | Email: tahara-minako@sec.nagoya-cu.ac.jp | | Date completed: 1 Aug 2025 | | |
| --- | --- | --- | --- | --- | --- | --- |
|  |  |  |  | | | |
|  | **1. TRANSLATION** |  |  | | | |
|  | | A -­‐No revisions | | | ■ |  |
|  | | B -­‐ Revision(s) suggested | | | ☐ |  |
|  | | C -­‐ Revision(s) required | | | ☐ |  |

If “B” or “C,” please provide an explanation or example:

**2. BOOLEAN AND PROXIMITY OPERATORS**

| A -­‐No revisions | ■ |
| --- | --- |
| B -­‐ Revision(s) suggested | ☐ |
| C -­‐ Revision(s) required | ☐ |

If “B” or “C,” please provide an explanation or example:

**3. SUBJECT HEADINGS**

| A -­‐No revisions | ■ |
| --- | --- |
| B -­‐ Revision(s) suggested | ☐ |
| C -­‐ Revision(s) required | ☐ |

If “B” or “C,” please provide an explanation or example:

**4. TEXT WORD SEARCHING**

| A -­‐No revisions | ■ |
| --- | --- |
| B -­‐ Revision(s)suggested | ☐ |
| C -­‐ Revision(s) required | ☐ |

If “B” or “C,” please provide an explanation or example:

**5. SPELLING, SYNTAX, AND LINE NUMBERS**

| A -­‐No revisions | ☐ |
| --- | --- |
| B -­‐ Revision(s)suggested | ■ |
| C -­‐ Revision(s) required | ☐ |

If “B” or “C,” please provide an explanation or example:

Cochrane CENTRAL Search Strings(Correction to some of brackets)

((autis* OR asperger* OR ("pervasive developmental disorders" OR "pervasive developmental disorder" OR "pervasive development disorders" OR "pervasive development disorder")):ti,ab,kw OR [mh "Child Development Disorders, Pervasive"]) AND ([mh "Sleep Wake Disorders"] OR (sleep* OR circadian*):ti,ab,kw) AND ([mh child] OR [mh Adolescent] OR [mh "Young Adult"] OR (child* OR adolescent* OR youth* OR (young NEXT adult*)):ti,ab,kw)

**6. LIMITS AND FILTERS**

| A -­‐No revisions | ■ |
| --- | --- |
| B -­‐ Revision(s) suggested | ☐ |
| C -­‐ Revision(s) required | ☐ |

If “B” or “C,” please provide an explanation or example:

OVERALL EVALUATION (Note: If one or more “revision required” is noted above, the response below must be “revisions required”.)

| A -­‐No revisions | ☐ |
| --- | --- |
| B -­‐ Revision(s) suggested | ■ |
| C -­‐ Revision(s) required | ☐ |

Additional comments:
